# miR-133b targets NCAPH to promote β-catenin degradation and reduce cancer stem cell maintenance in non-small cell lung cancer

**DOI:** 10.1038/s41392-021-00555-x

**Published:** 2021-07-07

**Authors:** Qiuxia Xiong, Liping Jiang, Kun Liu, Xiulin Jiang, Baiyang Liu, Yulin Shi, Dating Cheng, Yong Duan, Cuiping Yang, Yongbin Chen

**Affiliations:** 1 Department of Clinical Laboratory, the First Affiliated Hospital of Kunming Medical University, Kunming, China; 2 Key Laboratory of Animal Models and Human Disease Mechanisms of Chinese Academy of Sciences & Yunnan Province, Kunming Institute of Zoology, Kunming, Yunnan China; 3 Center for Excellence in Animal Evolution and Genetics, Chinese Academy of Sciences, Kunming, Yunnan China

**Keywords:** Cancer stem cells, Lung cancer

**Dear Editor**,

Non-small cell lung cancer (NSCLC), one of the most common lung cancers, is well known to have diverse pathological features. Cancer stem cells (CSCs) have been identified to play critical roles in tumor metastasis and drug resistance, while its potential clinical significance and molecular mechanism are still unclear. In addition, numerous findings have also shown that miRNAs play pivotal roles in CSCs during tumor progression. Our group recently identified NCAPH, which is one of the non-SMC regulatory components of condensing I complex involved in chromosome organization, as one of many pan-cancer biomarkers. However, how NCAPH is increased in NSCLC, as well as the underlying mechanism by which NCAPH promotes cancer progression, are poorly understood.

In this study, we found that NCAPH is highly expressed in NSCLC, whose high expression is gradually induced with increased TNM stage progression, and is associated with worse overall survival rate (Fig. 1a–d, Supplementary Fig. S1a–e, and Table S1). Furthermore, the mutation pattern of NCAPH in NSCLC was identified^1^ (Supplementary Fig. S1f and Table S2). In addition, we found that NCAPH knockdown inhibited cell proliferation, which can be rescued by NCAPH overexpression (Fig. 1e–h, Supplementary Fig. S1g–j and Table S3). We also examined the DNA synthesis by BrdU incorporation assay, and uncovered that BrdU positive cell populations, and the cell cycle regulators important for G0/G1 cell cycle transition, were dramatically reduced after NCAPH knockdown (Supplementary Fig. S1k–p).

**Fig. 1:**
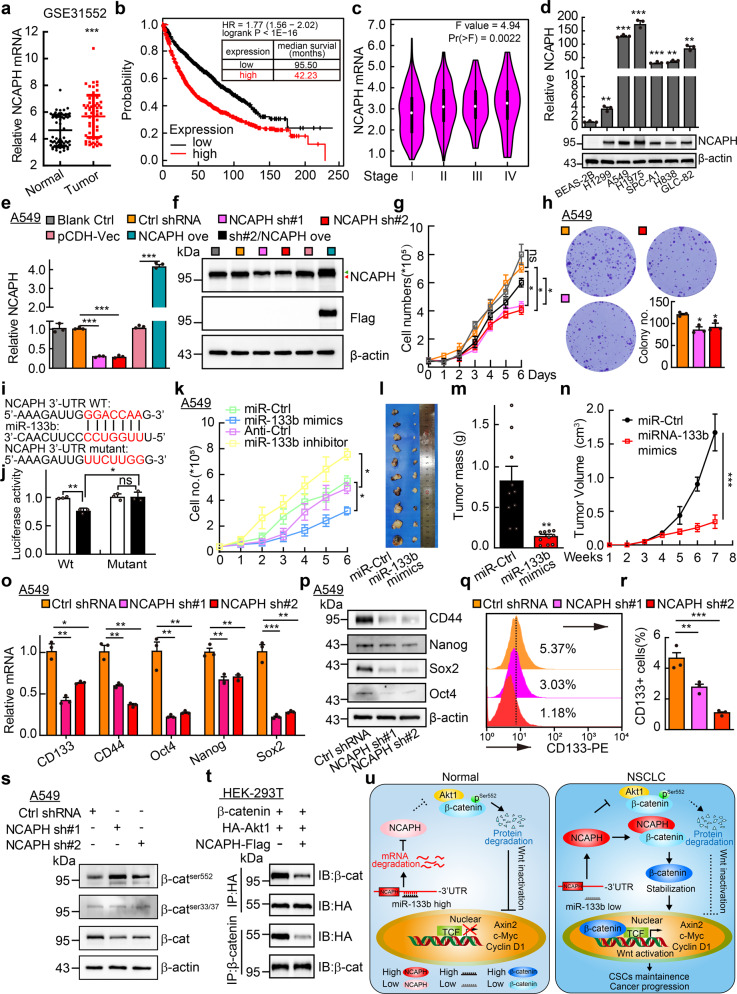
miR-133b targets NCAPH to promote β-catenin degradation and reduce cancer stem cell maintenance in NSCLC. **a** Significant differential expression of NCAPH between tumor and normal tissues from lung (GEO accession code: GSE31552). **b** NCAPH high expression in NSCLC leads to significant worse survival rate than its low expression patients by Kaplan–Meier survival analysis. **c** Expression levels of NCAPH in lung cancer patients with different disease stages. **d** Both NCAPH mRNA (top) and protein (bottom) were increased in NSCLC cell lines: H1299, A549, H1975, SPC-A1, H838, and GLC-82, compared to normal human bronchial epithelial cell line: BEAS-2B. **e, f** Establishing NCAPH knockdown and overexpression cell lines in A549, verified by Real-time RT-PCR (**e**) and western blot (**f**). Green arrow: exogenous NCAPH-Flag; red arrow: endogenous NCAPH. Gray box: Untreated blank control. **g, h** NCAPH knockdown in A549 dramatically inhibited cell proliferation (**g**) and colony formation (**h**), quantification data for (**h**) is also indicated. **i** Binding sites of NCAPH 3′-UTR sequences with miR-133b seed sequences predicted by TargetScanHuman. **j** miR-133b targets NCAPH verified by dual-luciferase reporter assay. **k** Overexpressions of miR-133b mimics or inhibitor suppressed or promoted, respectively, A549 cell proliferation determined by growth curve. **l** Xenograft tumors are photographed, which were harvested from A549 cells after tumors had grown for 8 weeks. **m, n** miR-133b overexpression significantly reduced xenograft tumor mass (**m**) and tumor volume (**n**) in nude mice. **o, p** NCAPH knockdown inhibited expressions of stemness marker genes in A549 examined by Real-time RT-PCR (**o**) and western blot (**p**). **q, r** NCAPH knockdown decreased membrane-tethered CD133 expression in A549 verified by flow cytometry analysis after stained with CD133-PE. (**r**) is the quantification of (**q**). **s** Indicated cell lysates were examined by western blot in A549 detecting phosphorylation on β-catenin. **t** To detect the complex formation among NCAPH, Akt1, and β-catenin, HEK-293T cells were transfected with indicated plasmids and the cell lysates were used for co-IP and western blot. **u** Working model for miR-133b/NCAPH axis in NSCLC. Under normal conditions, high level of miR-133b targets NCAPH mRNA and inhibits NCAPH expression. Decreased NCAPH proteins cannot inhibit Akt1/ β-catenin complex formation efficiently, resulting in phosphorylation at ser552 site on β-catenin leading to its protein degradation through ubiquitin proteasome pathway, which in turn reduces the activity of Wnt signaling pathway. However, under pathological conditions, decreased miR-133b promotes the high expression of NCAPH. Abundant NCAPH proteins compete with Akt1 to interact with β-catenin physically, which leads to stabilization of β-catenin and then constitutive activation of Wnt signaling. Therefore, the self-renewal ability of cancer stem cells in NSCLC is increased, which promotes NSCLC cancer progression and DDP resistance. NCAPH sh#1 = NCAPH shRNA#1, NCAPH sh#2 = NCAPH shRNA#2, NCAPH ove NCAPH overexpression, pCDH-vec pCDH vector. Data were shown as means ± SEM, *P < 0.05; **P < 0.01; ***P < 0.001; *t*-test

To figure out whether NCAPH knockdown induced cell cycle arrest leads to cellular apoptosis, which sensitizes cell responding to chemotherapy drug cisplatin (*cis*-Diamminedichloroplatinum, DDP), indicated cells were collected for annexin V staining followed by flow cytometry analysis, and higher percentages of apoptotic cell population in NCAPH knockdown groups either with or without DDP treatment were detected (Supplementary Fig. S2a–d). Tumor cell migration ability was also examined, which was dramatically inhibited after NCAPH knockdown (Supplementary Fig. S2e–k).

To uncover how NCAPH is increased in NSCLC, we applied the TargetScanHuman to predict the potential microRNAs directly targeting to NCAPH,^2^ and multiple candidate microRNAs were identified (Supplementary Table S4). To narrow down the exact functional miRNAs, we examined the expression levels of the candidate miRNAs in NSCLC using web source datasets,^3,4^ three miRNAs including miR-133b, miR-140, and miR-338 caught our attention because they are negatively associated with NCAPH expression and clinical outcome, and they are also markedly decreased in NSCLC. However, only miR-133b overexpression markedly inhibited NCAPH expression and tumor cell proliferation (Supplementary Fig. S3a–e). The direct 3′-UTR binding site of miR-133b in NCAPH mRNA was identified,^2^ and the luciferase activity of wild-type but not the mutant NCAPH 3′-UTR containing reporter construct was inhibited by miR-133b mimics overexpression (Fig. 1i, j). Consistently, the proliferation, cell survival, colony formation, and migration abilities of tumor cells were suppressed by miR-133b mimics, while were promoted by miR-133b inhibitor, which were reversed by NCAPH overexpression (Fig. 1k and Supplementary Figs. S3f–r, S4a–f).

To further test whether miR-133b/NCAPH axis plays important role during NSCLC progression in vivo, the xenograft tumor formation assay was performed. The xenograft tumor weights and volumes in miR-133b mimics forced expression A549 cells were inhibited (Fig. 1l–n and Supplementary Fig. S4g–m). Consistent with web source available dataset,^3^ we detected that miR-133b was decreased in the peripheral blood serum isolated from NSCLC patients (Supplementary Fig. S5a–c, and Table S5). In addition, we identified that NCAPH high expression in NSCLC positively correlates with the expressions of Sox2, CD44, ALCAM, and CD133, which were well characterized as lung cancer stem cell marker genes (Supplementary Fig. S5d, e). The existence of CSCs is one of the major causes for chemo- or radio-therapy resistance, together with the result that NCAPH knockdown promotes DDP induced cellular apoptosis, we hypothesized that miR-133b/NCAPH axis regulates the stemness maintenance of CSCs in NSCLC. Indeed, we found that the expressions of CD133, CD44, Oct4, Nanog, and Sox2 were all decreased (Fig. 1o–r and Supplementary Fig. S5f–i), and the tumor sphere numbers were dramatically decreased upon NCAPH depletion as well as under the serial diluted cell culture conditions (Supplementary Fig. S5j–m). Consistently, miR-133b mimics inhibited, while miR-133b inhibitor promoted, the expressions of CSCs marker genes and the tumor sphere formation ability (Supplementary Fig. S6a–o). In addition, the prognostic value of miR-133b or NCAPH in NSCLC was shown (Supplementary Fig. S6p).

To decipher the underlying mechanism, we applied KEGG analysis and web source available dataset again,^3^ and found that Wnt signaling was associated with miR-133b and NCAPH (Supplementary Fig. S7a, b). We then performed both the TOP Flash luciferase reporter and Real-time RT-PCR assays to examine the activity of canonical Wnt signaling pathway, and found that both the Wnt3a ligand and LiCl activated signaling as well as the downstream bona fide targets Axin2, c-Myc, and cyclin D1 were decreased upon NCAPH knockdown or miR-133b mimics overexpression (Supplementary Figs. S7c–f, S4k–m). We also examined β-catenin stability and the protein half-life by western blot upon NCAPH knockdown or miR-133b mimics overexpression, and marked reduced β-catenin proteins in both cytoplasmic and nucleus were detected, which was stabilized by proteasome inhibitor MG132 treatment (Supplementary Fig. S7g–l). As increasing evidence has shown that β-catenin proteins phosphorylated on Serin 552 site mediated by Akt1, Serin 33/37 sites mediated by GSK-3β, are preferentially degraded in a proteasome signaling pathway dependent manner, we therefore examined β-catenin phosphorylation levels on specific sites using western blot. We found that phosphorylation on Ser552 but not Ser33/37 was dramatically increased upon NCAPH knockdown (Fig. 1s and Supplementary Fig. S7m). In addition, we detected that endogenous NCAPH proteins interact with β-catenin proteins physically (Supplementary Fig. S7n, o), while the exogenous Akt1/β-catenin complex formation was abrogated by NCAPH overexpression (Fig. 1t and Supplementary Fig. S7p).

Accumulating evidence has indicated that dysfunctions of human condensins result in human cancers due to chromosomal instability. In line with former finding that miR-133b inhibits cell growth, migration, and invasion in NSCLC,^5^ we demonstrated that miR-133b downstream target NCAPH can compete with Akt1 to form a complex with β-catenin (Fig. 1u), which in turn inhibits β-catenin phosphorylation and ubiquitin mediated protein degradation, leading to activated Wnt signaling and a more viable niche for cancer stem cells in NSCLC. In summary, this is the first study to characterize the functional roles of miR-133b/NCAPH axis in lung cancer stem cells and NSCLC progression, which provides potential diagnostic and therapeutic biomarkers for NSCLC in the future.

## Supplementary information

Supplementary Materials
