# Supplementary material for: miR-133b targets NCAPH to promote β-catenin degradation and reduce cancer stem cell maintenance in non-small cell lung cancer
Source: Signal Transduct Target Ther. 2021 Jul 7;6:252. doi: 10.1038/s41392-021-00555-x (PMC8260594; doi:10.1038/s41392-021-00555-x)
Supplement: Supplementary file 1 — Supplementary Materials [file 41392_2021_555_MOESM1_ESM.doc]

Supplementary Materials for

**miR-133b targets NCAPH to promote β-catenin degradation and reduce cancer stem cell maintenance in non-small cell lung cancer**

Qiuxia Xiong^1, ‡^, Liping Jiang^2, ‡^, Kun Liu^2, ‡^, Xiulin Jiang^2^, Baiyang Liu^2^, Yulin Shi^2^, Dating Cheng^1^, Yong Duan^1,*^, Cuiping Yang^2,*^, Yongbin Chen^2,3, *^

1. Department of Clinical Laboratory, the First Affiliated Hospital of Kunming Medical University, Kunming, 650032, China.
2. Key Laboratory of Animal Models and Human Disease Mechanisms of Chinese Academy of Sciences & Yunnan Province, Kunming Institute of Zoology, Kunming, Yunnan 650223, China.
3. Center for Excellence in Animal Evolution and Genetics, Chinese Academy of Sciences, Kunming, Yunnan 650223, China.

‡ These authors contributed equally to this work.

^*^ Correspondence email: Yongbin Chen: [ybchen@mail.kiz.ac.cn](mailto:ybchen@mail.kiz.ac.cn)

Cuiping Yang : [cuipingyang@mail.kiz.ac.cn](mailto:cuipingyang@mail.kiz.ac.cn)

Yong Duan: [Duanyong7@139.com](mailto:Duanyong7@139.com)

**This PDF file includes:**

Materials and Methods

Figures. S1 to S7

Table S1 to S5

**Material and Methods**

**Cell culture**

The BEAS-2B cell line was purchased from cell bank of Kunming Institute of Zoology, and cultured in BEGM media (Lonza, CC-3170). HEK-293T was obtained from ATCC. Lung cancer cell lines, including A549, H1299, H1975, H838, GLC-82 and SPC-A1, were purchased from Cobioer, China with STR document, A549, H1299, H1975, H838, GLC-82 and SPC-A1 cells were all cultured in RPMI1640 medium (Corning) supplemented with 10% fetal bovine serum (FBS) and 1% penicillin/streptomycin. HEK-293T cells were cultured in DMEM medium (Corning).

**Constructs, transfection and infection**

The NCAPH-3×Flag plasmid was synthesized by Shanghai Generay Biotech. Akt1 was generated by RT-PCR and sub-cloned into pCDNA3.1 vector with a 3×HA tag at the N-terminus. Independent shRNAs targeting NCAPH were synthesized and cloned into the lentiviral plasmid pLKO.1 (Addgene, Cambridge, USA). The lenti-viruses were generated according to the manufacturer’s protocol. The NCAPH shRNAs, control scrambled shRNA or NCAPH-3×Flag were transfected into HEK-293T cells with the psPAX2/pMD2.G plasmids (Addgene) using calcium phosphate. After transfection, the cell supernatants were harvested and used to infect A549 and H1975 cells, and the stably lenti-viral infected cells were selected with puromycin. The miRNA control or miR-133b mimics, inhibitor, and miR-133b negative control were purchased from RiboBio. Cells were transfected with indicated miRNAs or control oligos using Lipofectamine 3000 (Invitrogen), and then collected for various assays.

**Immuno-precipitation, western blot and Real-time RT-PCR**

Cells were lysed in IP lysis buffer (1mM NaF, 50 mM Tris-HCl, pH 8.0, 120 mM NaCl, 0.5% NP40, 1 mM EDTA), supplemented with complete protease inhibitor cocktail (Complete Mini, Roche). To detect the physical interaction among NCAPH, β-catenin and Akt1 proteins, indicated constructs were transfected into HEK-293T cells, and indicated cell lysates were subjected to immunoprecipitation with indicated primary antibodies. The precipitated proteins were detected with indicated antibodies by western blot. For cycloheximide (CHX) or MG132 treatment assays, indicated cells treated with 100 μg/mL CHX, or 20 μM MG132 for 24 h, were harvested and lysed at indicated time points, and the cell lysates were subjected to western blot. For western blot analysis, proteins were resolved on SDS polyacrylamide gels, and then transferred to a polyvinylidene difluoride membrane. After blocking with 5% (w/v) milk, the membrane was stained with indicated primary antibodies. For Real-time RT-PCR assay, indicated cells were lysed by RNAiso Plus (Takara Bio, Beijing, China, Cat# 108-95-2). Total RNA was extracted according to the manufacturer’s protocol, and then reverse transcribed using RT reagent Kit (Takara Bio, Beijing, China, Cat# RR047A; TIANGEN Biotech, Beijing, China, Cat# KR211-02). Real-time PCR was performed by FastStart Universal SYBR Green Master Mix (Roche, Cat# 04194194001; TIANGEN Biotech, Beijing, China, Cat# FP411-02) using an Applied Biosystems 7500 machine. The primers and antibodies used in this study are shown in **Table S3**.

**Cell proliferation, BrdU incorporation, colony formation, tumor sphere formation assays**

For cell proliferation assay, indicated cells were plated into 12-well plates at a density of 2×10^4^, the cell numbers were subsequently counted each day using an automatic cell analyzer countstar (Shanghai Ruiyu Biotech Co., China, IC 1000). For BrdU incorporation assay, cells were cultured in 8-well plates for 24h, pulsed with 10 μM BrdU (Abcam, Cat# ab142567) for 20 min, and fixed with 4% PFA (paraformaldehyde). Cells were then incubated with BrdU (Cell Signaling Technology, Cat# 5292s, dilution 1:1000) primary antibody followed by secondary antibody detection (Abclonal, Cat# 61303, dilution 1:500). Cell nuclei were stained with DAPI (4',6-diamidino-2-phenylindole). For colony formation assay, indicated cells were seeded in 6-well plate with 500 cells per well supplemented with 2 mL cell culture medium, and the cell culture medium was changed every 3 days for 2~3 weeks. Indicated cells were fixed with 4% PFA and stained with 0.5% crystal violet. For tumor sphere formation assay, indicated cells were plated in ultralow-attachment 6 well plates (Corning; 3471), cultured in serum-free DMEM/F12 supplemented with B27, 20 ng/mL EGF and 20 ng/mL bFGF, and 4 μg/mL heparin. 14 days after culture, the spheres were pictured and counted.

**Xenograft tumor formation assay**

Male nude mice aged 4-6 weeks were subcutaneously injected with A549 cell lines (1×10^6^ cells), when the xenograft tumors reached to 50 mm^3^ of volume, they were randomly divided into two groups. The mice were injected with miR Ctrl or mimics (5 nM) around the tumor twice per week. Four weeks later, all mice were sacrificed at the end of the experiment and the tumors were harvested and weighted. Nude mice were monitored every other day, xenograft tumor weights and volumes were measured with a sliding caliper, and tumor volumes were calculated using the formula (L×W^2^)/2. All animals were kept in a SPF environment and the protocols were pre-approved and conducted under the policy of Animal care and Use Committee at the Kunming Institute of Zoology, CAS.

**Dual-luciferase assay**

Putative binding sites for miR-133b on the 3’-UTR of NCAPH were predicted by Targetscan Version 7.2 software ([http://www.targetscan.org](http://www.targetscan.org/)). The identified DNA fragment was subcloned into pmiRglo plasmid (Promega) using the following primer: Forward Primer: 5’-AAAGAGCTCCACAGGTGAGACTCCACACTCTGT-3’; Reverse Primer: 5’-CCAAGCTTGGCTCAAGTGATTATCCCACCTCA-3’. HEK-293T cells (2×10^4^ cells/well) were seeded in a 24-well plate and co-transfected with 3’-UTR NCAPH construct and miR-133b mimics or miR Ctrl using Lipofectamine 3000. Both firefly and Renilla luciferase expressions were measured post-transfection using the Dual Luciferase Kit (Promega) according to the manufacturer’s instructions.

**Immunohistochemical staining (IHC)**

For immunohistochemical staining, the sections were deparaffinized in xylene and rehydrated through graded ethanol. Antigen retrieval was performed for 20 min at 95 °C with sodium citrate buffer (pH 6.0). After quenching endogenous peroxidase activity with 3% H_2_O_2_ and blocking non-specific binding with 1% bovine serum albumin buffer, sections were incubated overnight at 4°C with indicated primary antibodies. Following several washes, the sections were treated with HRP conjugated secondary antibody for 40 min at room temperature, and stained with 3, 3-diaminobenzidine tetrahydrochloride (DAB). Slides were photographed with microscope (Olympus BX43F, Japan). The photographs were analyzed based on the ratio of the staining with the Image-Pro Plus 7.0 software (Media Cybernetics, Inc., Silver Spring, MD, USA).

**Subcellular fractionation**

For nuclear and cytoplasmic fractionations, cells were washed twice with cold PBS and collected, followed by the addition of lysis buffer A (20 mM Tris (pH 7.5), 20% glycerol, 10mM NaCl, 1.5mM MgCl_2_, 5mM EDTA (pH 8.0), 1% NP-40, and a proteinase inhibitor cocktail; Roche). Cell lysates were lysed on ice for 10 min, followed by centrifugation at 800 × g for 4min at 4 °C. The supernatant was transferred (cytoplasmic extract) to a clean pre-chilled tube. The pellet was sequentially washed 3 times with lysis buffer A and then lysed in buffer B (20mM Tris(pH 7.5), 10% glycerol, 100mM NaCl, 1.5mM MgCl_2_, 5mM EDTA (pH 8.0), 1mM DTT, 1% NP-40 and a proteinase inhibitor cocktail) for 30 min on ice, followed by centrifugation at 9,000 × rpm at 4 °C for 10 min. The supernatants were collected as the nuclear fractions.

**Cell migration assays**

To produce a wound, the monolayer cells in 6-well plate were scraped in a straight line with pipette tips. Plate was then washed with warm PBS to remove detached cells. Photographs of the scratch were taken at indicated time points using Nikon inverted microscope (Ti-S). Gap width was calculated with GraphPad Prism software. For trans-well assay, 1-2×10^4^ cells in 100 μL serum free medium were plated in an 8.0-μm, 24-well plate chamber insert (Corning Life Sciences, catalog no. 3422), with medium containing 10% FBS at the bottom of the insert. Cells were incubated for 24 h, and then fixed with 4% paraformaldehyde for 20 min. After washing, cells were stained with 0.5% crystal violet blue. The positively stained cells were examined under the microscope.

**Bioinformatic analysis**

All datasets used in this study were available to the public. Expression of miRNAs, mRNAs in Gene Expression Omnibus (GEO) and TCGA dataset were obtained from the GEO website [^45^](#_ENREF_45), TCGA official website [^46^](#_ENREF_46) and starBase [^29^](#_ENREF_29), the datasets were analyzed by GEO2R. The survival analysis was performed according to the GEPIA website [^47^](#_ENREF_47), KM plotter [^48^](#_ENREF_48) and OncomiR dataset [^49^](#_ENREF_49). KEGG pathway enrichment analysis was performed using the “clusterProfiler” package in R [^50^](#_ENREF_50).

**Statistical analysis**

The significance of the data between two experimental groups was determined by Student’s t-test, and multiple group comparisons were analyzed by one-way ANOVA. *P* < 0.05 (*), *P* < 0.01 (**) and *P* < 0.001 (***), were considered significant.

**Author contributions**

Y.B.C., C.P.Y and Y.D. supervised the study. Y.B.C and C.P.Y wrote the manuscript. Q.X.X. identified and performed the functional analysis for miR-133b in NSCLC. Q.X.X., L.P.J., and K.L. designed and performed the functional analysis for miR-133b/NCAPH axis in NSCLC. X.L.J., B.Y.L., Y.L.S., and D.T.C. helped and performed the in vivo xenograft tumor formation assay and bioinformatic analysis.

**Figure. S1.**

**
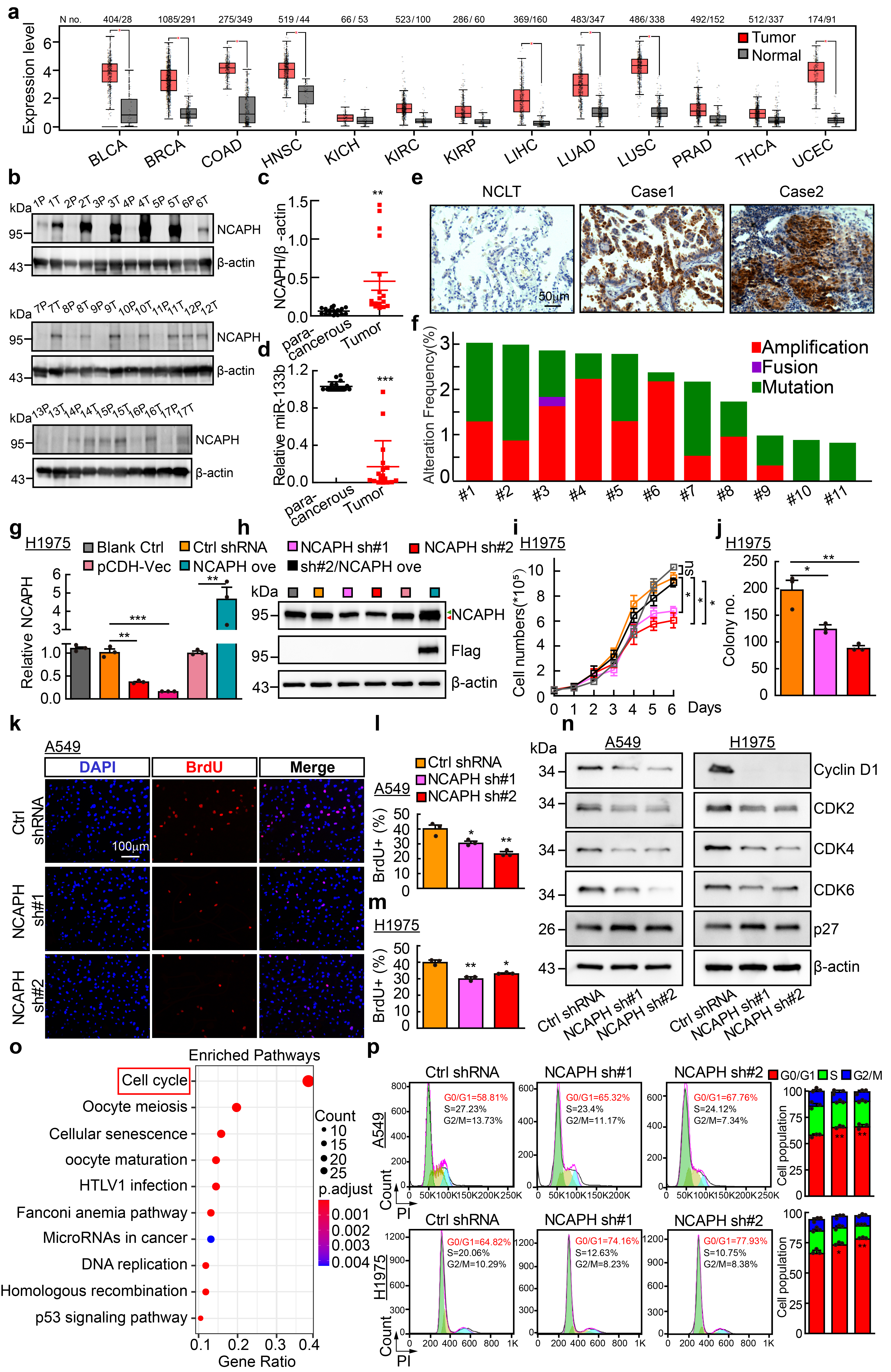
**

**Figure. S1.** NCAPH regulates tumor cell proliferation in NSCLC. **a** NCAPH expression is highly expressed in various types of human cancers using GEPIA (Gene Expression Profiling Interactive Analysis). Red box: Tumor tissues; Grey box: Normal tissues. **b, c** NCAPH is highly expressed in NSCLC cancerous tissues examined by western blot. P: paracancerous tissue, T: tumor. (**c**) is the quantification data for (**b**). The numbers indicated different tissues. **d** The expression of miR-133b was examined by Real-time RT-PCR. **e** NCAPH expression is highly expressed in NSCLC cancerous tissues examined by IHC staining. NCLT: non cancerous lung tissue. Case1 and case2 were NSCLC cancerous tissues. **f** NCAPH is frequently mutated in lung cancer. Mutation: green; amplification: red; fusion: purple. **g, h** Establishing NCAPH knockdown and overexpression cell lines in H1975, verified by Real-time RT-PCR (**e**) and western blot (**f**). Green arrow: exogenous NCAPH-Flag; red arrow: endogenous NCAPH. Grey box: untreated blank control. **i-m** NCAPH knockdown dramatically inhibited H1975 cell proliferation (**i**) and colony formation abilities (**j**). (**k**) is the representative images for BrdU incorporation assay in indicated cells, and (**l, m**) are quantification data for BrdU incorporation assays in A549 and H1975, respectively. **n** NCAPH knockdown regulated the expressions of cell cycle regulators in A549 and H1975 examined by western blot. **o** NCAPH related signaling pathways in NSCLC were enriched by Kyoto Encyclopedia of Genes and Genomes (KEGG) pathway analysis. Oocyte maturation: progesterone-mediated oocyte maturation. HTLV1 infection: human T-cell leukemia virus type 1 infection. **p** NCAPH knockdown induced cell cycle arrest at G0/G1 phase in A549 (top panel) and H1975 (bottom panel). Quantification data for cell cycle analysis in A549 (top panel) and H1975 (bottom panel), respectively, were also indicated. Data are shown as means±SEM, * *P* <0.05; ** *P* <0.01; *** *P* <0.001; *t*-test.

**Figure. S2.**

**
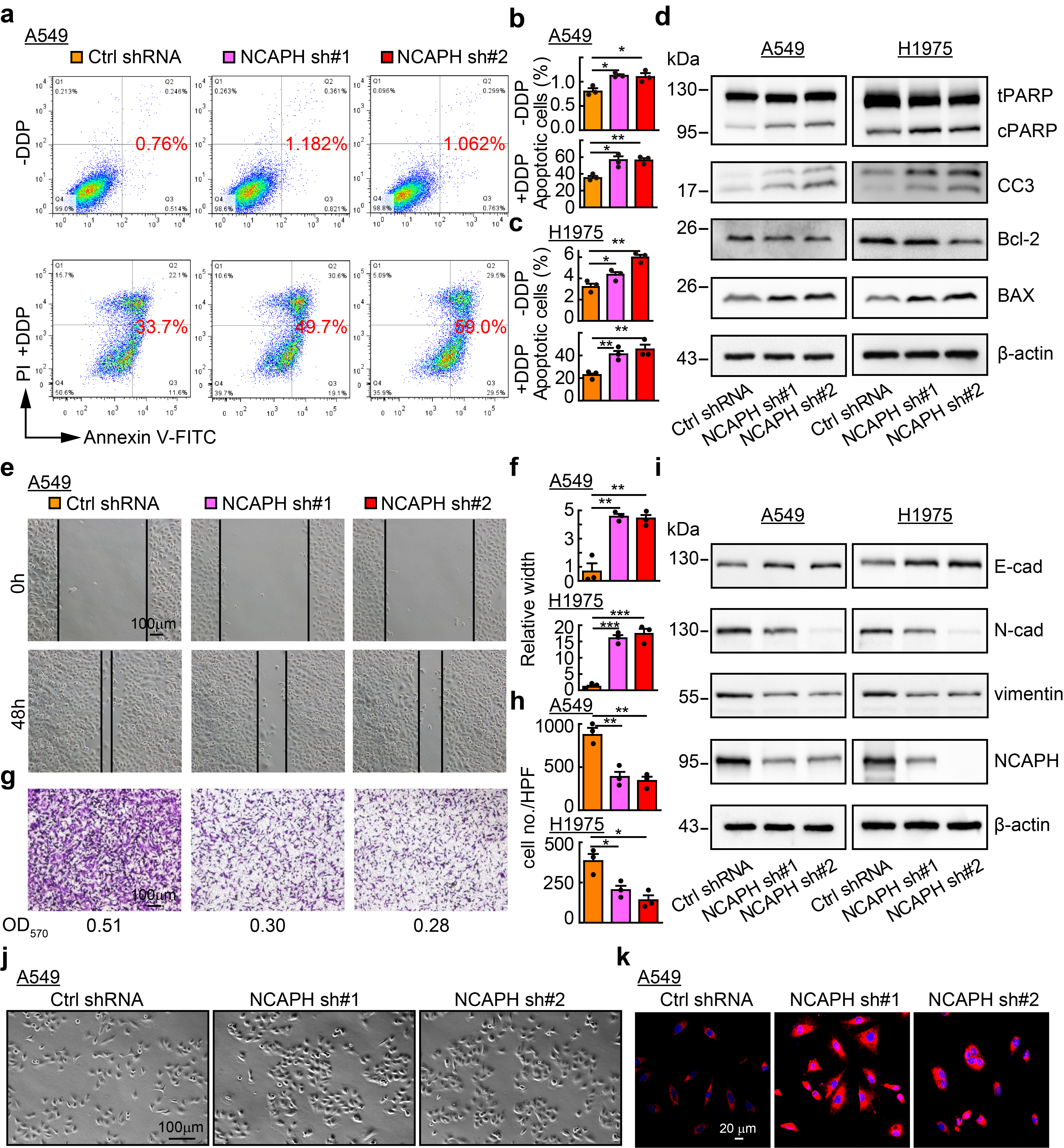
**

**Figure. S2.** NCAPH knockdown inhibits cell survival and cell migration. **a-c** NCAPH knockdown promotes cellular apoptosis with (bottom) or without (top) cisplatin (DDP) treatment in A549 cells. Cells were treated with 25 μM DDP, and were stained with Annexin V/PI followed by flow cytometry analysis (**a**). **b, c** Quantification data for cellular apoptosis with or without DDP in indicated cells. **d** NCAPH inhibition promotes cellular apoptosis in A549 and H1975 examined by western blot using indicated antibodies. **e, f** Knockdown of NCAPH inhibited cell migration using wound healing assay in A549 and H1975. (**f**) is the quantification data. **g, h** Knockdown of NCAPH inhibited A549 cell migration using trans-well assay. (**h**) is quantification data. **i** NCAPH regulates the expressions of cell migration related factors in A549 and H1975 cells examined by western blot. **j** Representative phase-contrast images showing the morphology of the indicated cell. **k** Representative immunostaining images of E-cadherin (red) and DAPI (blue) for the indicated cells. Data are shown as means±SEM, * *P* <0.05; ** *P* <0.01; *** *P* <0.001; *t*-test.

Figure. S3.

**
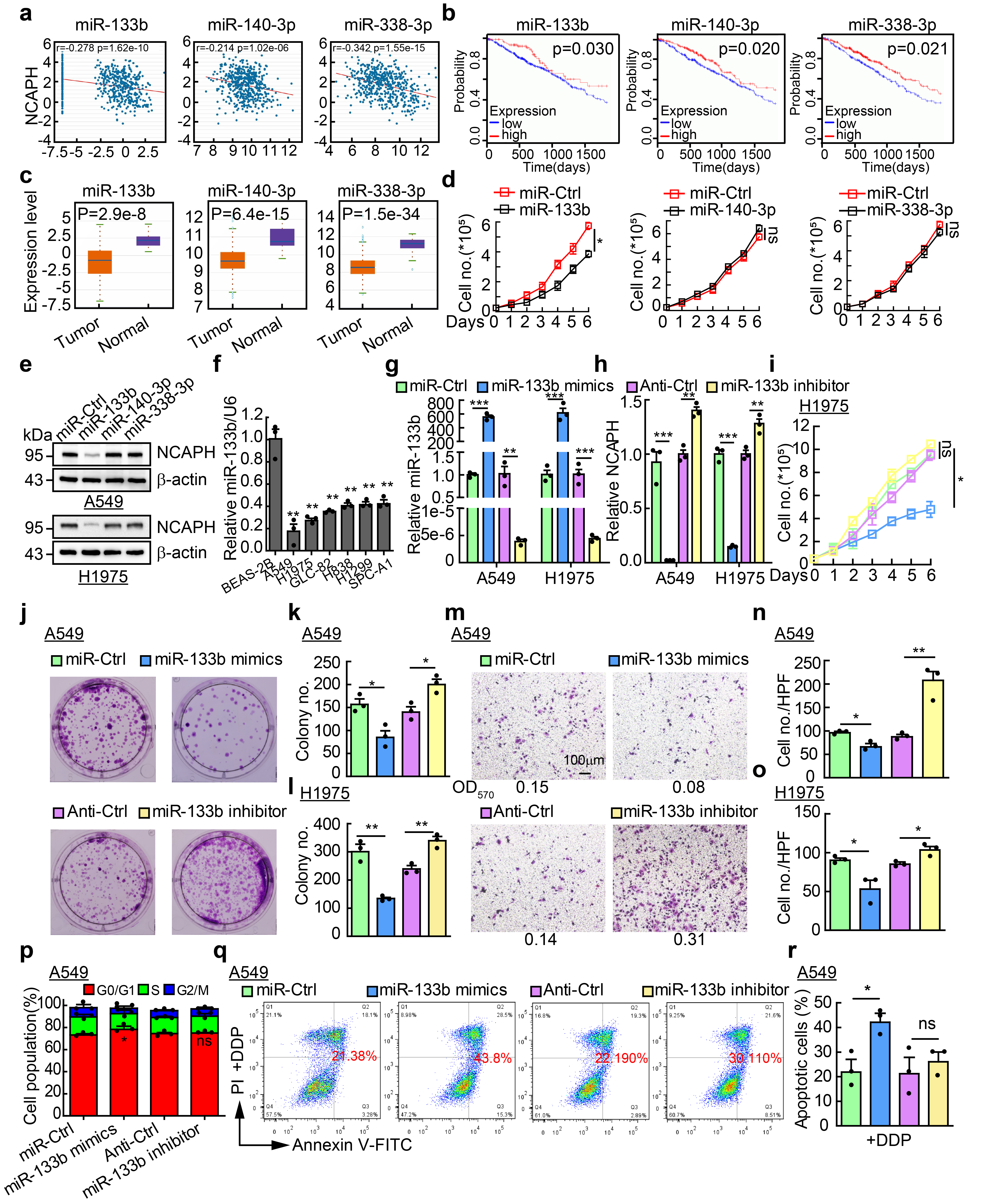
**

**Figure. S3.** miR-133b targets NCAPH in NSCLC. **a** NCAPH is negatively associated with miR-133b, miR-140-3p and miR-338-3p using starBase dataset. **b** Low expressions of miR-133b, miR-140-3p and miR-338-3p correlate with worse survival time using OncomiR dataset. **c** The expressions of miR-133b, miR-140-3p and miR-338-3p are decreased in lung tumor tissues compared with normal group using starBase dataset. **d** The effects of miR-133b miR-140-3p and miR-338-3p mimics overexpressions on A549 cell proliferation by growth curve assay. **e** Examining NCAPH protein expression after miR-133b, miR-140-3p or miR-338-3p mimics overexpression in A549 or H1975 cells, respectively, by western blot. **f** miR-133b is decreased in NSCLC cancerous cell lines compared with BEAS-2B by Real-time RT-PCR. **g, h** The relative expression levels of miR-133b (**g**) and NCAPH (**h**) transcripts detected by Real-time RT-PCR. **i** Forced expressions of miR-133b mimics or inhibitor suppressed or promoted, respectively, H1975 cell proliferation. **j-l** Forced expressions of miR-133b mimics or inhibitor suppressed or promoted, respectively, colony formation ability in A549 and H1975. (**k, l)** are quantification data for colony formation in A549 and H1975, respectively. **m-o** Forced expressions of miR-133b mimics or inhibitor repressed or promoted, respectively, A549 cell migration by trans-well assay. (**n, o)** are quantification data for trans-well assay in A549 and H1975, respectively. The OD_570_ numbers were also indicated below for indicated images. **p** Forced expression of miR-133b mimics induced cell cycle arrest at G0/G1 phase in A549 cells. Quantification data for cell cycle analysis is shown. **q-r** Forced expression of miR-133b mimics promoted cellular apoptosis with 25 μM cisplatin (DDP) treatment in A549 cells. Indicated cells were stained with Annexin V/PI followed by flow cytometry analysis (**q**). **r** Quantification data for (q).

**Figure. S4.**

**
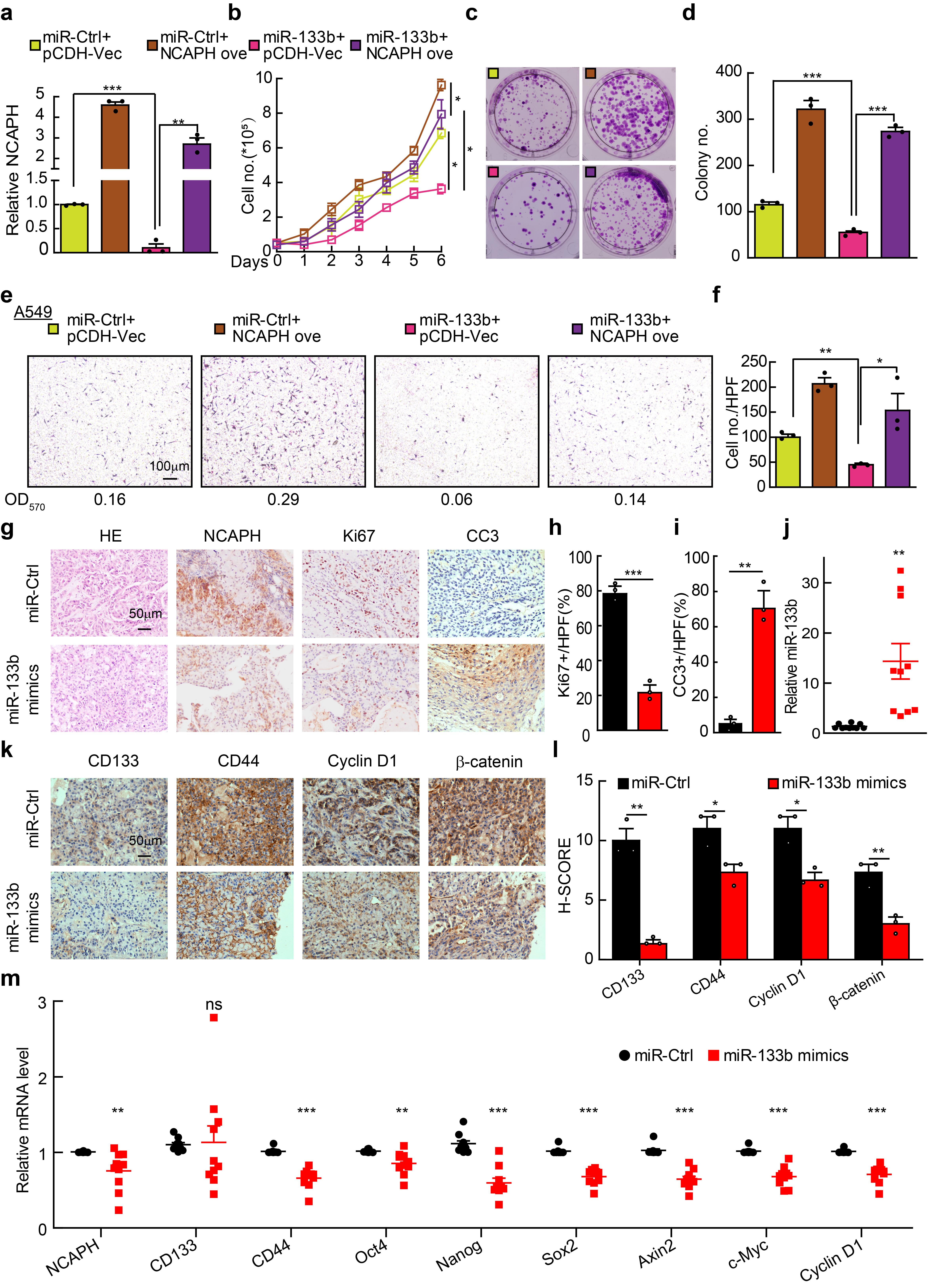
**

**Figure. S4.** miR-133b targets NCAPH to regulate NSCLC progression. **a-f** Overexpression of NCAPH reversed miR-133b mimics overexpression induced phenotypes, including cell growth (**a, b**), colony formation (**c, d**) and trans-well (**e, f**) abilities. Quantification data were indicated. **g-i** Representative images of hematoxylin and eosin, and IHC staining in xenograft tissues using indicated primary antibodies. **h, i** Quantification data for Ki67 (**h**) and CC3 (**i**) in (**g**). **j** The expression of miR-133b in indicated xenograft tumors examined by Real-time RT-PCR. **k** Representative images of IHC staining in indicated xenograft tumors using indicated primary antibodies. **l** Quantification data for (**k**). **m** Indicated gene expressions were examined by Real-time RT-PCR using indicated xenograft tumors. CC3=cleaved caspase 3. Data are shown as means±SEM, * *P* <0.05; ** *P* <0.01; *** *P* <0.001; *t*-test.

**Figure. S5.**

**
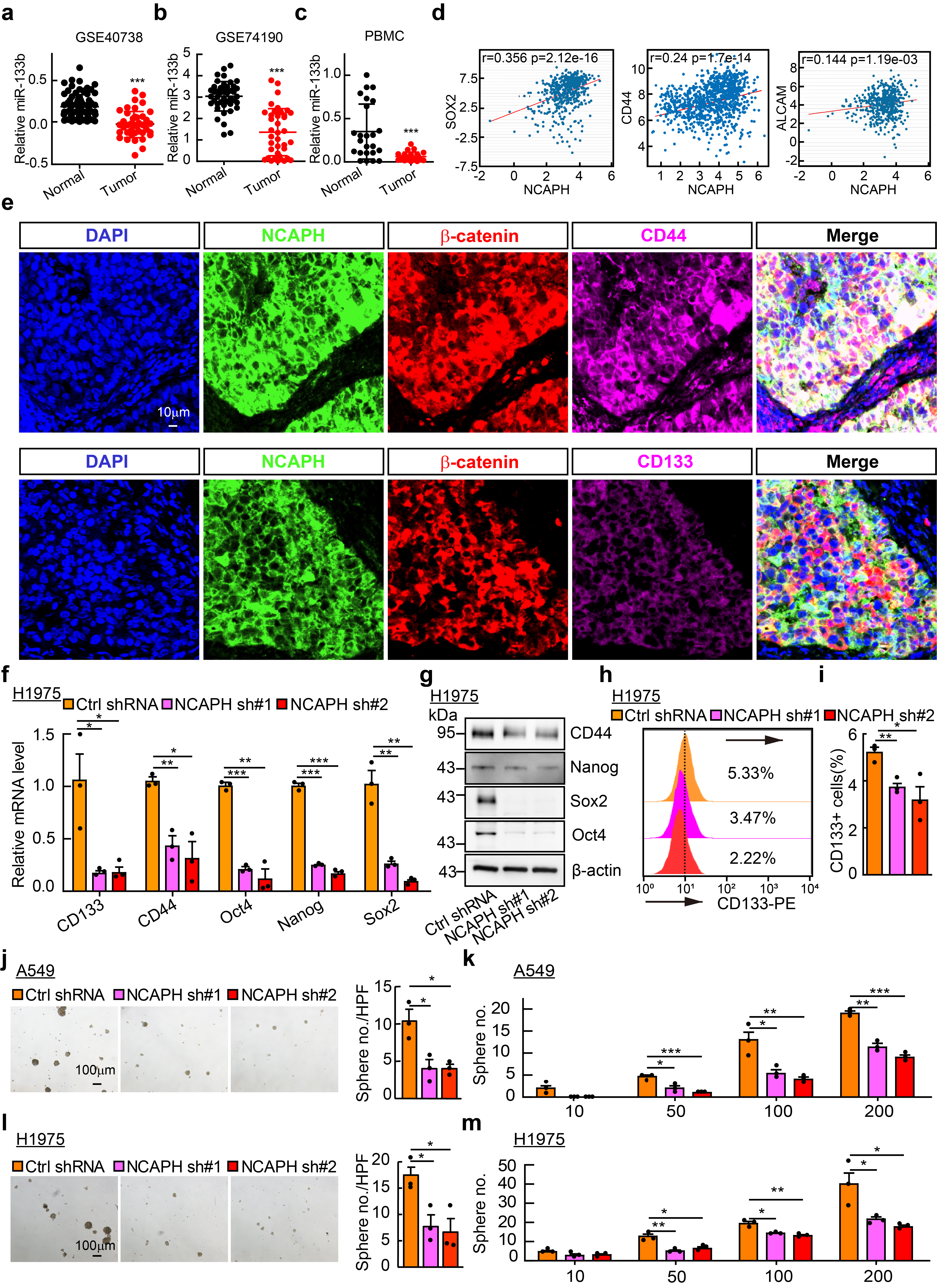
**

**Figure. S5.** NCAPH promotes cancer stem cell maintenance in NSCLC. **a-b** Significant differential expression of miR-133b between normal and tumor groups in NSCLC, including peripheral blood (GEO accession code: GSE40738) (**a**) and tissues (GEO accession code: GSE74190) (**b**). **c** Examining miR-133b expression level in fresh peripheral blood serum samples isolated from normal and NSCLC patients, respectively, verified by Real-time RT-PCR. **d** NCAPH is positively associated with lung cancer stem cell marker genes: Sox2 and ALCAM (CD166) analyzed by starBase dataset, CD44 analyzed by GEPIA database. **e** The colocalization of NCAPH, β-catenin, CD133 and CD44 in cancerous tissues examined by immunostaining using indicated antibodies. **f, g** NCAPH knockdown inhibited expressions of stemness marker genes in H1975 examined by Real-time RT-PCR (**f**) and western blot (**g**). **h, i** NCAPH knockdown decreased membrane-tethered CD133 expression in H1975 verified by flow cytometry analysis after stained with CD133-PE. (**i**) is the quantification for (**h**). **j** Representative images of tumor spheres stably expressing ctrl shRNA and NCAPH shRNAs in A549. The quantification data for (**j**) is also indicated. **k** NCAPH knockdown decreased cancer stem cell self-renewal ability using in vitro ELDA (Extreme limiting dilution analysis) in A549. **l** Representative images of tumor spheres stably expressing ctrl shRNA and NCAPH shRNAs in H1975. The quantification data for (**l**) is also indicated. **m** NCAPH knockdown decreased cancer stem cell self-renewal ability using in vitro ELDA (Extreme limiting dilution analysis) in H1975. Data are shown as means±SEM, * *P* <0.05; ** *P* <0.01; *** *P* <0.001; *t*-test.

**Figure. S6.**

**
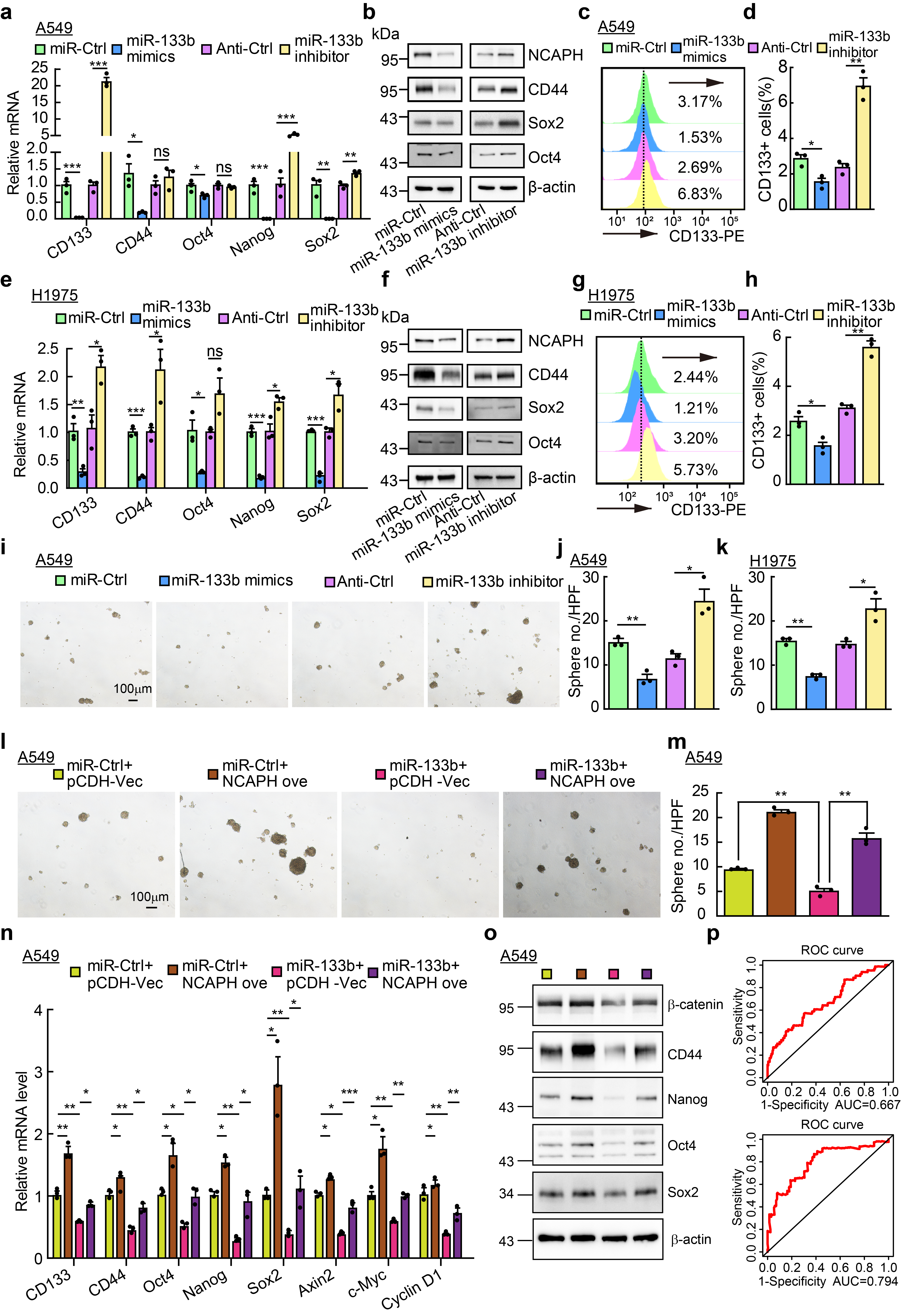
**

**Figure. S6.** Overexpression of miR-133b inhibits cancer stem cell maintenance in NSCLC. **a, b** Forced expressions of miR-133b mimics or inhibitor suppressed or promoted, respectively, expressions of stemness marker genes in A549 examined by Real-time RT-PCR (**a**) and western blot (**b**). **c, d** miR-133b mimics or inhibitor decreased or increased, respectively, membrane-tethered CD133 expression in A549 verified by flow cytometry analysis. (**d**) is the quantification for (**c**). **e, f** Forced expressions of miR-133b mimics or inhibitor suppressed or promoted, respectively, expressions of stemness marker genes in H1975 examined by Real-time RT-PCR (**e**) and western blot (**f**). **g, h** miR-133b mimics or inhibitor decreased or increased, respectively, membrane-tethered CD133 expression in H1975 verified by flow cytometry analysis. (**h**) is the quantification for (**g**). **i-k** Representative images of tumor spheres expressing indicated oligos in A549. (**j, k**) is the quantification data for tumor spheres in A549 and H1975, respectively. **l, m** Overexpression of NCAPH reversed miR-133b mimics overexpression-decreased tumor sphere formation ability. (**m**) is the quantification data for (**l**). **n, o** Overexpression of NCAPH reversed miR-133b mimics overexpression phenotype examined by Real-time RT-PCR (**n**) and western blot (**o**). **p** ROC curve for NCAPH (AUC=0.667) and miR-133b (AUC=0.794) in LUAD using TCGA dataset. The area under the ROC curve >0.5 was considered to have a certain diagnostic value. At the same time, the closer the area under the ROC curve to 1, the higher the diagnostic value of miR-133b and NCAPH for the diagnosis of NSCLC. Data are shown as means±SEM, * *P* <0.05; ** *P* <0.01; *** *P* <0.001; *t*-test.

Figure. S7.


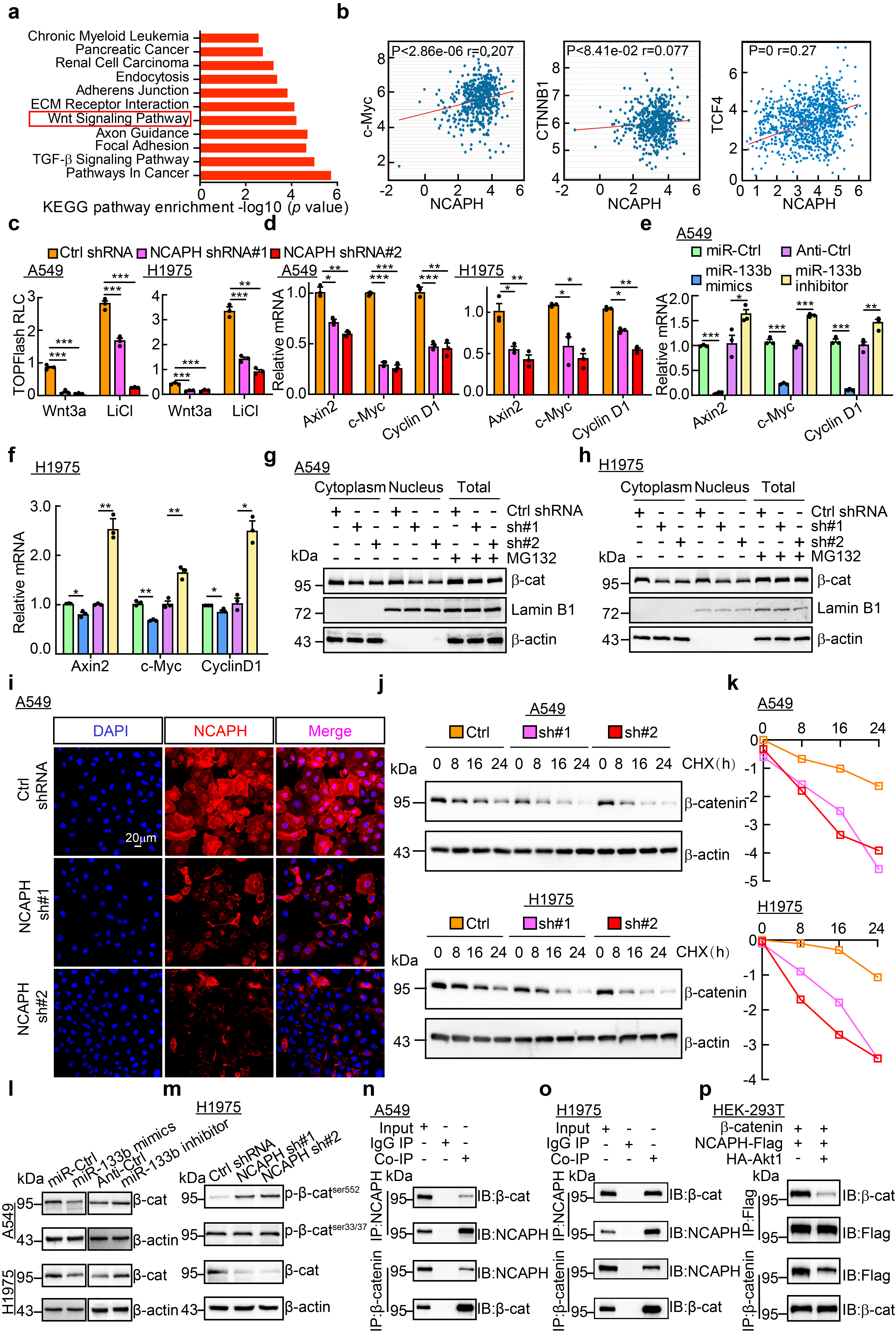


**Figure. S7.** NCAPH stabilizes β-catenin proteins and activates Wnt signaling in NSCLC. **a** miR-133b related signaling pathway is enriched by KEGG analysis in NSCLC. Pluripotency of stem cells: signaling pathways regulating pluripotency of stem cells. Oocyte maturation: progesterone-mediated oocyte maturation. **b** NCAPH is positively associated with stem cell marker genes including Myc, CTNNB1 (β-catenin) and TCF4 analyzed by starBase dataset. **c** NCAPH knockdown inhibited Wnt signaling pathway activity using TOPFlash reporter assay in A549 and H1975. **d** NCAPH knockdown reduced expressions of Wnt signaling downstream genes in A549 and H1975 detected by Real-time RT-PCR. **e, f** Relative expression of Wnt signaling downstream genes in cells expressing indicated oligos detected by Real-time RT-PCR in A549 (**e**) and H1975 (**f**). **g, h** NCAPH knockdown reduced the stability of β-catenin proteins in A549 (**g**) and H1975 (**h**), which was reversed MG132 (20 μM) treatment examined by western blot. Lamin B: nuclear fraction; β-actin: cytosol fraction. **i** Representative immunostaining images showing the cellular distribution of β-catenin proteins in indicated cells. **j-k** Lysates of indicated cell lines treated by cycloheximide (CHX: 100 μg/mL) were analyzed by western blot with indicated primary antibodies. (**k**) is the quantification data for (**j**). **l** Forced expressions of miR-133b mimics or inhibitor suppressed or promoted, respectively, expressions of β-catenin proteins in A549 and H1975 examined by western blot. **m** Indicated cell lysates were examined by western blot detecting  phosphorylation on β-catenin. **n, o** Co-IP assay to detect the endogenous protein interaction between NCAPH and β-catenin in A549 and H1975. Cell lysates were immunoprecipitated with indicated antibodies and analyzed by western blot. **p** To detect the complex formation among NCAPH, Akt1 and β-catenin, HEK-293T were transfected with indicated plasmids and the cell lysates were used for co-IP and western blot. Data are shown as means±SEM, * *P* <0.05; ** *P* <0.01; *** *P* <0.001; *t*-test.

**Table S1. The full name of cancer types shown in Figure S1a.**

| **Abbreviation** | **Full name of cancer** |
| --- | --- |
| BLCA | Bladder urothelial carcinoma |
| BRCA | Breast invasive carcinoma |
| COAD | Colon adenocarcinoma |
| HNSC | Head and Neck squamous cell carcinoma |
| KICH | Kidney Chromophobe |
| KIRC | Kidney renal clear cell carcinoma |
| KIRP | kidney renal papillary cell carcinoma |
| LIHC | Liver hepatocellular carcinoma |
| LUAD | Lung adenocarcinoma |
| LUSC | Lung squamous cell carcinoma |
| PRAD | Prostate adenocarcinoma |
| THCA | Thyroid carcinoma |
| UCEC | Uterine Corpus Endometrial Carcinoma |

**Table S2. The mutation information for NCAPH in lung cancer.**

| **SampleID** | **Cancer study** | **CancerTyPe** | **A change** | **Type** |
| --- | --- | --- | --- | --- |
| LUAD-S01315 | Lung Adenocarcinoma (Broad, Cell 2012) | LungAdenocarcinoma | D551H | Missense |
| LUAD-YINHD | Lung Adenocarcinoma (Broad, Cell 2012) | LungAdenocarcinoma | E573K | Missense |
| LUAD-NYU284 | Lung Adenocarcinoma (Broad, Cell 2012) | Lung.Adenocarcinoma | P5555 | Missense |
| BGI-RS36 | Lung Adenocarcinoma (OncoSG, Nat Genet 2020) | LungAdenocarcinoma | T487A | Missense |
| BGI-RS36 | Lung Adenocarcinoma (OncoSG, Nat Genet 2020) | LungAdenocarcinoma | C714F | Missense |
| BCI-WC10 | Lung Adenocarcinoma (OncoSG, Nat Genet 2020) | LungAdenocarcinoma | P23L | Missense |
| TCGA-44-3919-01 | Lung Adenocarcinoma (TCGA, Firehose Legacy) | LungAdenocarcinoma | L724V | Missense |
| TCGA-49-4514-01 | Lung Adenocarcinoma (TCGA, Firehose Legacy) | LungAdenocarcinoma | G321W | Missenso |
| TCGA-75-5126-01 | Lung Adenocarcinoma (TCGA, Firehose Legacy) | LungAdenocarcinoma | Q626* | Nonsense |
| TCGA-91-6829-01 | Lung Adenocarcinoma (TCGA, Firehose Legacy) | LungAdenocarcinoma | D94E | Missense |
| TCGA-49-4514-01 | Lung Adenocarcinoma (TCGA, PanCancer Atlas) | LungAdenocarcinoma | G321W | Missense |
| TCGA-75-5126-01 | Lung Adenocarcinoma (TCGA, PanCancer Atlas) | LungAdenocarcinoma | Q626* | Nonsense |
| TCGA-91-6829-01 | Lung Adenocarcinoma (TCGA, PanCancer Atlas) | LungAdenocarcinoma | D94E | Missense |
| TCGA-44-2656-01 | Lung Adenocarcinoma (TCGA, PanCancer Atlas) | LungAdenocarcinoma | K152R | Missenso |
| TCA-44-A4S4U-01 | Lung Adenocarcinoma (TCGA, PanCancer Atlas) | LungAdenocarcinoma | K597E | Missense |
| TCGA-50-6590-01 | Lung Adenocarcinoma (TCGA, PanCancer Atlas) | LungAdenocarcinoma | A534S | Missense |
| TCA-55-7910-01 | Lung Adenocarcinoma (TCGA, PanCancer Atlas) | LungAdenocarcinoma | Q601H | Missense |
| TCGA-97-8175-01 | Lung Adenocarcinoma (TCGA, PanCancer Atlas) | LungAdenocarcinoma | M4231 | Missense |
| TCGA-97-A4M7-01 | Lung Adenocarcinoma (TCGA, PanCancer Atlas) | LungAdenocarcnoma | P2920ts*3 | FSdel |
| TCGA-17-Z026-01 | Lung Adenocarcinoma (TCGA, PanCancer Atlas) | Lung.Adenocarcinoma | G610R | Missense |
| TCGA-73-4662-01 | Lung Adenocarcinoma (TCGA, PanCancer Atlas) | LungAdenocarcinoma | Q143H | Missense |
| TCGA-73-4666-01 | Lung Adenocarcinoma (TCGA, PanCancer Atlas) | LungAdenocarcinoma | X7_splice | Splice |
| TCA-44-3919-01 | Lung Adenocarcinoma (TCGA, Nature 2014) | LungAdenocarcinoma | L724V | Missense |
| TCGA-49-4514-01 | Lung Adenocarcinoma (TCGA, Nature 2014) | LungAdenocarcinoma | G321W | Missense |
| TCGA-75-5126-01 | Lung Adenocarcinoma (TCGA, Nature 2014) | LungAdenocarcinoma | Q626* | Nonsense |
| TCG4-91-6829-01 | Lung Adenocarcinoma (TCGA, Nature 2014) | Lung Adenocarcinoma | D94E | Missense |
| TCGA-22-5473-01 | Lung Squamous Cell Carcinoma (TCGA, Firehose Legacy) | Lung Squamous Cell Carcinoma | 1315M | Missense |
| TCGA-22-5473-01 | Lung Squamous Cell Carcinoma (TCGA, PanCancer Atlas) | Lung Squamous Cell Carcinoma | 315M | Missense |
| TCGA-37-3792-01 | Lung Squamous Cell Carcinoma (TCGA, PanCancer Atlas) | Lung Squamous Cell Carcinoma | w327C | Missense |
| TCGA-39-5011-01 | Lung Squamous Cell Carcinoma (TCGA, PanCancer Atlas) | Lung squamous Cell Carcinoma | D443A | lssense |
| TCGA-98-A531-01 | Lung Squamous Cell Carcinoma (TCGA, PanCancer Atlas) | Lung squamous Cell Carcinoma | T4351 | Missense |
| TCGA-18-3406-01 | Lung Squamous Cell Carcinoma (TCGA, PanCancer Atlas) | Lung squamous cell carcinoma | w327L | Missense |
| TCGA-22-5473-01 | Lung Squamous Cell Carcinoma (TCGA, Nature 2012) | Lung squamous Cell Carcinoma | 1315M | Missense |
| LUAD-S01315-Tumor | Pan-Lung Cancer (TCGA, Nat Genet 2016) | Lung Adenocarcinoma | D551H | issense |
| LUAD-YINHD-Tumar | Pan-Lung Cancer (TCGA, Nat Genet 2016) | Lung Adenocarcinoma | E573K | Missense |
| TCGA-44-3919-01 | Pan-Lung Cancer (TCGA, Nat Genet 2016) | Lung Adenocarcinoma | L724V | Missense |
| TCGA-49-4514-01 | Pan-Lung Cancer (TCGA, Nat Genet 2016) | Lung Adenocarcinoma | G321w | Missense |
| TCGA-75-5126-01 | Pan-Lung Cancer (TCGA, Nat Genet 2016) | Lung Adenocarcinoma | 626* | Nonsense |
| TCGA-91-6829-01 | Pan-Lung Cancer (TCGA, Nat Genet 2016) | Lung Adonocarcinoma | D94E | Mlssense |
| TCGA-22-5473-01 | Pan-Lung Cancer (TCGA, Nat Genet 2016) | Lung squamous Cell Carcinoma | 1315M | Missenso |
| TCGA-37-3792-01 | Pan-Lung Cancer (TCGA, Nat Genet 2016) | Lung squamous Cell Carcinoma | w327C | Missense |
| TCGA-39-5011-01 | Pan-Lung Cancer (TCGA, Nat Genet 2016) | Lung Squamous Cell Carcinorma | D443A | Missense |
| TCGA-44-2656-01 | Pan-Lung Cancer (TCGA, Nat Genet 2016) | Lung Adenocarcinoma | K152R | Missense |
| TCGA-44-A4SU-01 | Pan-Lung Cancer (TCGA, Nat Genet 2016) | Lung Adenocarcinoma | K597E | Missense |
| TCGA-50-6590-01 | Pan-Lung Cancer (TCGA, Nat Genet 2016) | Lung Adenocarcinoma | A534S | Missense |
| TCGA-55-7910-01 | Pan-Lung Cancer (TCGA, Nat Genet 2016) | Lung Adenocarcinoma | Q601H | Missense |
| TCGA-97-8175-01 | Pan-Lung Cancer (TCGA, Nat Genet 2016) | Lung Adenocarcinoma | M4231 | Mssense |
| TCGA-97-44N17-O1 | Pan-Lung Cancer (TCGA, Nat Genet 2016) | Lung Adenocarcinoma | p292Qfs*3 | FS del |
| TCGA-98-453L-01 | Pan-Lung Cancer (TCGA, Nat Genet 2016) | Lung squamous Cell carcinoma | T4351 | Missense |
| TCGA-51-6867-01 | Pan-Lung Cancer (TCGA, Nat Genet 2016) | Lung squamous Cell Carcinoma | Q535L | Missense |
| CRUKO016-R1 CRUK0016-R2 | Non-Small Cell Lung Cancer (TRACERx, NEJM & Nature 2017) | Non-SmalCellLungCancer | M137L | Missense |
|  | Non-Small Cell Lung Cancer (TRACERx, NEJM & Nature 2017) | Non-SmallCellLungCancer | M137L | Missense |
| CRUKO090-R1 CRUK0090-R2 TCGA-90-7767-01 | Non-Small Cell Lung Cancer (TRACERx, NEJM & Nature 2017) | LungSquamousCellCarcinoma | F431L | Missense |
|  | Non-Small Cell Lung Cancer (TRACERx, NEJM & Nature 2017) | LungSquamousCellCarcinoma | F431L | Missonso |
|  | Lung Squamous Cell Carcinoma (TCGA, PanCancer Atlas) | LungSquamousCellCarcinoma | NCAPH.KCNIP3 | Fusion |
| Small Cell Lung Cancer (U Cologne, Nature 2015) | sclc_ucologne_2015_S02344 | Small Cell Lung Cancer | T119N | Missense |

| **Cancer type** | **Cancerstudy** | **DOI** | **Journal** |
| --- | --- | --- | --- |
| #1 | Lung Adenocarcinoma (TCGA, Nature 2014) | doi: 10.1038/nature13385 | Nature |
| #2 | Lung Adenocarcinoma (TCGA, PanCancer Atlas) | TCGA |  |
| #3 | Lung Squamous Cell Carcinoma (TCGA, PanCancer Atlas) | TCGA |  |
| #4 | Lung Squamous Cell Carcinoma (TCGA, Nature 2012) | doi: 10.1038/nature11404. | Nature |
| #5 | Pan-Lung Cancer (TCGA, Nat Genet 2016) | doi: 10.1038/ng.3564. | Nat Genet. |
| #6 | Lung Squamous Cell Carcinoma (TCGA, Firehose Legacy) | TCGA Provisional. |  |
| #7 | Lung Adenocarcinoma (Broad, Cell 2012) | doi: 10.1016/j.cell.2012.08.029. | Cell |
| #8 | Lung Adenocarcinoma (TCGA, Firehose Legacy) | TCGA Provisional. |  |
| #9 | Lung Adenocarcinoma (OncoSG, Nat Genet 2020) | doi: 10.1038/s41588-019-0569-6. | Nat Genet. |
| #10 | Non-Small Cell Lung Cancer (TRACERx, NEJM) | DOI: 10.1056/NEJMoa1616288 | N Engl J Med |
| #10 | Non-Small Cell Lung Cancer (Nature 2017) | DOI: 10.1038/nature22364 | Nature |
| #11 | Small Cell Lung Cancer (U Cologne, Nature 2015) | doi: 10.1038/nature14664. | Nature |

**Table S3. Antibodies and primers used in the manuscript.**

| **Antibody Name** | **Catalog number** | **Dilution** | **Supplier** | **Species** |
| --- | --- | --- | --- | --- |
| CDK2 | 10122-1-AP | 1:2000 | Proteintech | Rabbit |
| CDK4 | ab108357 | 1:1000 | abcam | Rabbit |
| CDK6 | ab124821 | 1:2000 | abcam | Rabbit |
| Cyclin D1 | 60186-1-1g | 1:1000 | Proteintech | Mouse |
| β-actin | 60008-1-1g | 1:5000 | Proteintech | Mouse |
| p27 | 610241 | 1:2000 | BD | Mouse |
| E-cadherin | ab40772 | 1:500 | abcam | Rabbit |
| N-cadherin | ab18203 | 1:1000 | abcam | Rabbit |
| Vimentin | 103661-1-AP | 1:2000 | Proteintech | Rabbit |
| PARP | 9542S | 1:1000 | CST | Rabbit |
| Cleaved caspase3 | 9661S | 1:500 | CST | Rabbit |
| Bcl-2 | 15071S | 1:500 | CST | Mouse |
| Bax | ab77566 | 1:1000 | abcam | Mouse |
| β-catenin | 610153 | 1:2000 | BD | Mouse |
| NCAPH | 11515-1-AP | 1:1000 | Proteintech | Rabbit |
| Oct4 | ab19857 | 1:1000 | abcam | Rabbit |
| Sox2 | 23064S | 1:1000 | CST | Rabbit |
| Nanog | ab109250 | 1:1000 | abcam | Rabbit |
| HA | sc-7392 | 1:1000 | santa cruz | Mouse |
| Flag | F1804 | 1:1000 | Sigma | Mouse |
| Flag | 14793S | 1:500 | CST | Rabbit |
| CD44 | 3570s | 1:1000 | CST | Mouse |

| **Primer Name** | **Primer sequences(5'-3')** |
| --- | --- |
| Human β-actin_F | AAGTGTGACGTGGACATCCGC |
| Human β-actin_R | CCGGACTCGTCATACTCCTGCT |
| Human CD133_F | ATGGCAACAGCGATCAAGG |
| Human CD133_R | GTACTTTGTTGGTGCAAGCTCT |
| Human Sox2_F | CACAGATGCAACCGATGCA |
| Human Sox2_R | GGTGCCCTGCTGCGAGTA |
| Human CD44_F | CTGCCGCTTTGCAGGTGTA |
| Human CD44_R | CATTGTGGGCAAGGTGCTATT |
| Human Oct4_F | CTGGGTTGATCCTCGGACCT |
| Human Oct4_R | CCATCGGAGTTGCTCTCCA |
| Human Nanog_F | TTTGTGGGCCTGAAGAAAACT |
| Human Nanog_R | AGGGCTGTCCTGAATAAGCAG |
| Human NCAPH_F | AAACACGCAGATTACGGAACA |
| Human NCAPH_R | GTTGGTTGGTTCGGTGTCTTT |
| Human Axin2_F | AGTCAGCAGAGGGACAGGAA |
| Human Axin2_R | CTTCGTACATGGGGAGCACT |
| Human c-Myc_F | GGCTCCTGGCAAAAGGTCA |
| Human c-Myc_R | CTGCGTAGTTGTGCTGATGT |
| Human CyclinD1_F | GCTCCTGTGCTGCGAAGT |
| Human CyclinD1_R | TGTTCCTCTCAGACCTCCAG |
| Human NCAPH sh#1 Forward oligos | CCGGTCAGAGATTCTTAAACAGAAACTCGAGTTTCTGTTTAAGAATCTCTGATTTTTG |
| Human NCAPH sh#1 Reverse oligos | AATTCAAAAATCAGAGATTCTTAAACAGAAACTCGAGTTTCTGTTTAAGAATCTCTGA |
| Human NCAPH sh#2 Forward oligos | CCGGTCTCCTAAATTGATCTGTTATCTCGAGATAACAGATCAATTTAGGAGATTTTTG |
| Human NCAPH sh#2 Reverse oligos | AATTCAAAAATCTCCTAAATTGATCTGTTATCTCGAGATAACAGATCAATTTAGGAGA |
| Hsa-miR-133b_qPCR | TTTGGTCCCCTTCAACCAGCTA |
| Human U6_qPCR | CTCGCTTCGGCAGCACA |

**Table S4. The candidate miRNAs targeting NCAPH. Red: low expression; Blue: high expression.**

|  | **miRNA** | **Position in the UTR** | **seed match** | **context++ score** | **context++ score percentile** | **weighted context++ score** | **conserved branch length** | **Pct** |
| --- | --- | --- | --- | --- | --- | --- | --- | --- |
| 1 | hsa-miR-133b | 1574-1580 | 7mer-m8 | -0.12 | 71 | -0.01 | 0.173 | < 0.1 |
| 2 | hsa-miR-140-3p | 1976-1982 | 7mer-m8 | -0.09 | 76 | -0.01 | 0.262 | < 0.1 |
| 3 | hsa-miR-338-3p | 701-707 | 7mer-1A | -0.02 | 47 | 0 | 2.199 | < 0.1 |
| 4 | hsa-miR-1976 | 23-29 | 7mer-m8 | -0.16 | 90 | -0.16 | 0 | N/A |
| 5 | hsa-miR-6892-5p | 35-41 | 7mer-m8 | -0.08 | 61 | -0.08 | 0 | N/A |
| 6 | hsa-miR-495-3p | 130-136 | 7mer-m8 | -0.1 | 95 | -0.1 | 0.018 | N/A |
|  |  |  |  |  |  |  |  |  |
|  | **miRNA** | **Position in the UTR** | **seed match** | **context++ score** | **context++ score percentile** | **weighted context++ score** | **conserved branch length** | **Pct** |
| 1 | hsa-miR-493-5p | 408-414 | 7mer-m8 | -0.23 | 96 | -0.23 | 4.413 | N/A |
| 2 | hsa-miR-628-5p | 16-22 | 7mer-m8 | -0.21 | 93 | -0.21 | 0 | N/A |
| 3 | hsa-miR-93-3p | 17-23 | 7mer-m8 | -0.12 | 81 | -0.12 | 0 | N/A |
| 4 | hsa-miR-370-3p | 21-28 | 8mer | -0.34 | 98 | -0.34 | 1.014 | N/A |
| 5 | hsa-miR-660-3p | 24-30 | 7mer-m8 | -0.09 | 68 | -0.09 | 0.082 | N/A |
| 6 | hsa-miR-503-3p | 93-99 | 7mer-1A | -0.08 | 63 | -0.08 | 0 | N/A |
| 7 | hsa-miR-548v | 102-108 | 7mer-m8 | -0.06 | 48 | -0.06 | 0 | N/A |
| 8 | hsa-miR-3065-5p | 131-137 | 7mer-m8 | -0.05 | 57 | -0.05 | 0 | N/A |
| 9 | hsa-miR-1276 | 137-143 | 7mer-m8 | -0.19 | 95 | -0.19 | 0.01 | N/A |
| 10 | hsa-miR-185-5p | 147-154 | 8mer | -0.5 | 99 | -0.5 | 0.161 | N/A |
| 11 | hsa-miR-500b-3p | 162-168 | 7mer-m8 | -0.1 | 88 | -0.1 | 0.082 | N/A |
| 12 | hsa-miR-3127-5p | 174-181 | 8mer | -0.28 | 93 | -0.28 | 0.01 | N/A |
| 13 | hsa-miR-34a-3p | 195-201 | 7mer-1A | -0.1 | 84 | -0.1 | 0 | N/A |
| 14 | hsa-miR-545-5p | 205-211 | 7mer-m8 | -0.1 | 80 | -0.1 | 0 | N/A |
| 15 | hsa-miR-18a-3p | 239-246 | 8mer | -0.33 | 97 | -0.33 | 0.082 | N/A |
| 16 | hsa-miR-324-3p | 240-246 | 7mer-1A | -0.13 | 81 | -0.13 | 0.088 | N/A |
| 17 | hsa-miR-6802-3p | 248-254 | 7mer-m8 | -0.22 | 88 | -0.22 | 0 | N/A |
| 18 | hsa-miR-624-5p | 256-262 | 7mer-1A | -0.21 | 82 | -0.21 | 0 | N/A |

**Table S5. Clinicopathological characteristics of patients with non-small cell lung cancer (NSCLC) patients and health donor in the blood samples.**

| Patients characteristics | No. (%) |
| --- | --- |
| **NSCLC patients** |  |
| **Age(years)** |  |
| ≤50 | 19(80%) |
| ＞50 | 5(20%) |
| **Gender** |  |
| Male | 13(55%) |
| Female | 11(45%) |
| **Health donor** |  |
| **Age(years)** |  |
| ≤50 | 10(42.5%) |
| ＞50 | 14(57.5%) |
| **Gender** |  |
| Male | 7(30%) |
| Female | 17(70%) |

**Ethics Statement**

Samples were obtained with informed consent and all protocols were approved by The Second Xiangya Hospital of Central South University Ethics Review Board (Scientific and Research Ethics Committee, S-02/2000). Written informed consent was obtained from all patients，also the written informed consent was obtained from the next of kin, caretakers, or guardians on the behalf of the minors/children participants involved in your study.
